# A Rare Familial Case of Pseudohypoparathyroidism Type 1b in Two Brothers Presenting With Recurrent Leg Cramps and Learning Difficulties

**DOI:** 10.7759/cureus.99606

**Published:** 2025-12-19

**Authors:** Hishaam A Yunas, Awab Ismail, Ehtesam A Chowdhury, Rashid Riaz, Muzammil Hussain, Syeda S Amjad, Jayashekara Acharya

**Affiliations:** 1 Diabetes and Endocrinology, Hereford County Hospital, Hereford, GBR

**Keywords:** endocrinology, hypocalcemia, metabolic medicine, parathyroid hormone, pseudohypoparathyroidism

## Abstract

We present a rare familial case of two brothers presenting with late-onset symptoms of posturing, cramps, and occasional falls. The brothers, who were initially under the pediatric team being investigated for rare thiamine-transporter-related genetic disorders, had significant symptomatic hypocalcemia and cerebral calcifications identified on brain imaging. They were both diagnosed with pseudohypoparathyroidism type 1b in their late teenage years and achieved stable calcium levels and symptom control after treatment titration. This case highlights the importance of identifying key hypocalcemia symptoms such as fatigue, muscle cramps, and paresthesia, which may have led to earlier recognition of the condition.

## Introduction

Pseudohypoparathyroidism (PHP) is a rare endocrine disorder marked by the body's resistance to parathyroid hormone (PTH). This results in low calcium levels (hypocalcemia), high phosphate levels (hyperphosphatemia), and elevated PTH levels. The kidneys and bones fail to respond properly, leading to significant clinical and biochemical issues [1].

PHP can pose serious risks, including seizures, neuromuscular irritability, and chronic intracranial calcifications [2,3]. Patients may exhibit symptoms such as fatigue, muscle cramps, paresthesia, developmental delays, and, in some cases, features of Albright’s hereditary osteodystrophy (AHO) [2]. Affecting about one in 100,000 individuals, PHP is often diagnosed in childhood or adolescence, especially when physical or neurological signs appear, or when unexplained hypocalcemia is present [4]. While genetics are important for diagnosis, metabolic and drug-related causes are also key [5].

In PHP, there are over 400 genetic mutations implicated with the GNAS gene (encoding for G-protein-coupled receptor, a key part of the signaling pathway for parathyroid and other hormone action at their receptors) located on chromosome 20q13, with this being the primarily affected gene [2]. Based on the nature of the mutation, PHP can occur in conjunction with Albright’s hereditary osteodystrophy and multi-hormone resistance (types 1a and 1c), or with isolated renal tubular resistance only (type 2) [4,6]. In type 1b, because phenotypic abnormalities are absent, presentation may be delayed and therefore prove diagnostically challenging to identify. Due to this, similar case reports of type 1b have been reported late with atypical presentations.

One example of an atypical presentation was reported by Naganuma et al., in which a seven-year-old girl presented with seizures due to intracerebral calcifications as the index presentation of PHP type 1b. In another case reported by Ebrahim et al., a patient was not diagnosed until the age of 33 years with symptomatic hypocalcemia [7,8]. This study discusses the diagnostic journey of a patient initially misdiagnosed with a thiamine transporter or mitochondrial disorder.

## Case presentation

We present a 15-year-old male with a background of learning difficulties, undescended testes, and accelerated growth who was diagnosed in 2017 with pseudohypoparathyroidism after being admitted with extreme tiredness and fatigue. Despite having mild learning difficulties, he had normal developmental milestones and school performance. He had initially reported a nine-month history of recurrent leg cramps, abnormal toe posturing, tingling below the knees, and frequent episodes of sudden leg weakness resulting in occasional falls. Due to progressive worsening of these symptoms, concerns for a potential neuromuscular or metabolic disorder were raised.

**Figure 1:**
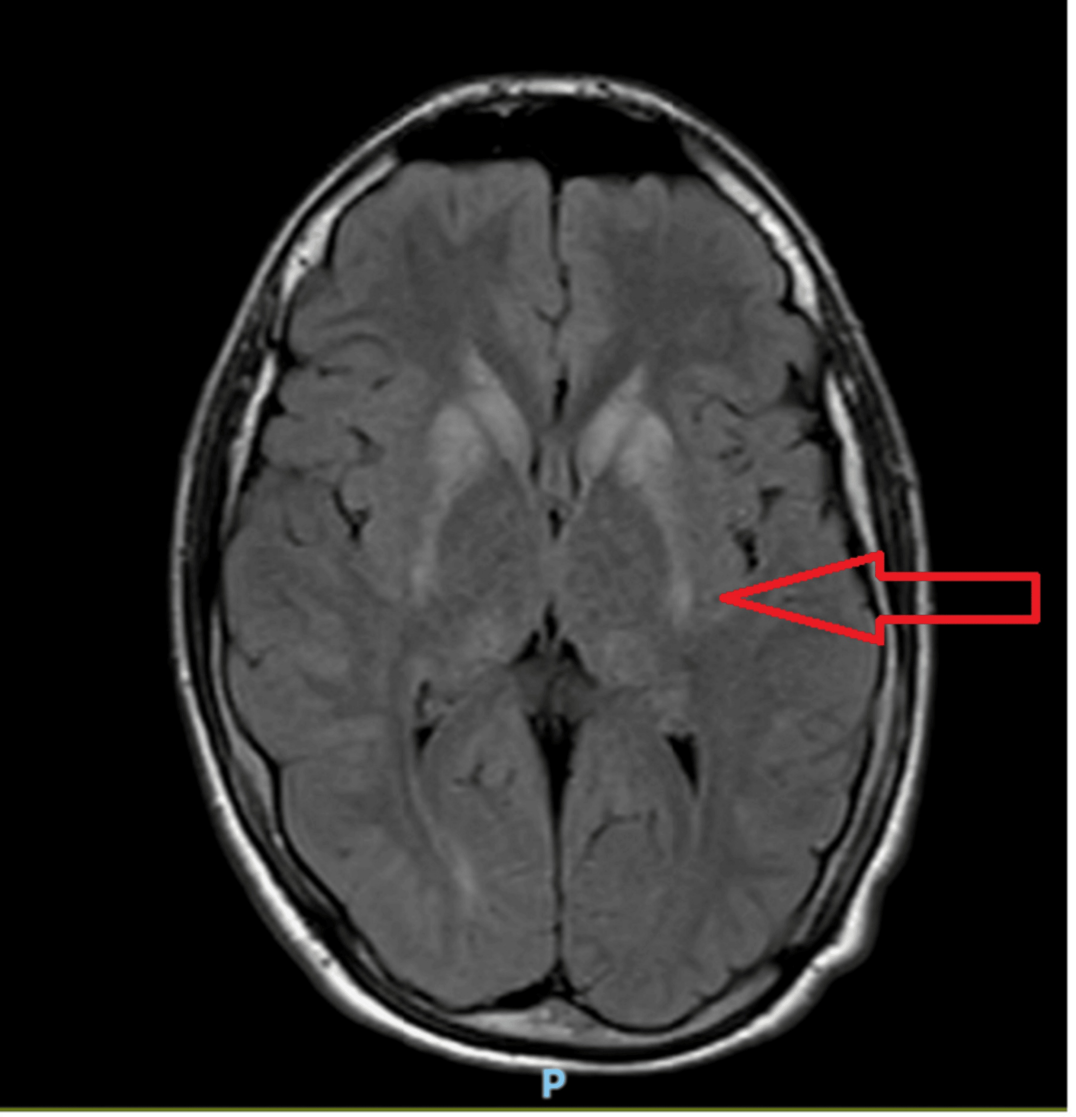
Axial FLAIR MRI of the brain demonstrating symmetrical signal changes in the bilateral basal ganglia and dentate nuclei, consistent with possible calcification (red arrow), one of the characteristic features of PHP. PHP: pseudohypoparathyroidism; FLAIR: fluid-attenuated inversion recovery

Initial examination did not demonstrate focal neurological deficits. However, a brain magnetic resonance imaging (MRI) was requested to exclude neurological causes of the aforementioned symptoms (Figure 1). A referral to the neurometabolic clinic was also made, where he was seen by both a pediatric neurologist and a genetic consultant. The MRI revealed symmetrical bilateral calcification of the basal ganglia and dentate nuclei. With the patient’s background and symptoms, this prompted suspicion of a thiamine transporter disorder, requiring genetic testing. He was prescribed daily thiamine 300 mg and biotin 20 mg in the interim, while awaiting genetic testing results. There was no notable clinical improvement with this. A mitochondrial disorder was also suspected, thus prompting baseline bone profile blood investigation to be undertaken; with a low corrected calcium (1.28 mmol/L) and magnesium (0.64 mmol/L), he was admitted to hospital for intravenous calcium therapy.

**Figure 2:**
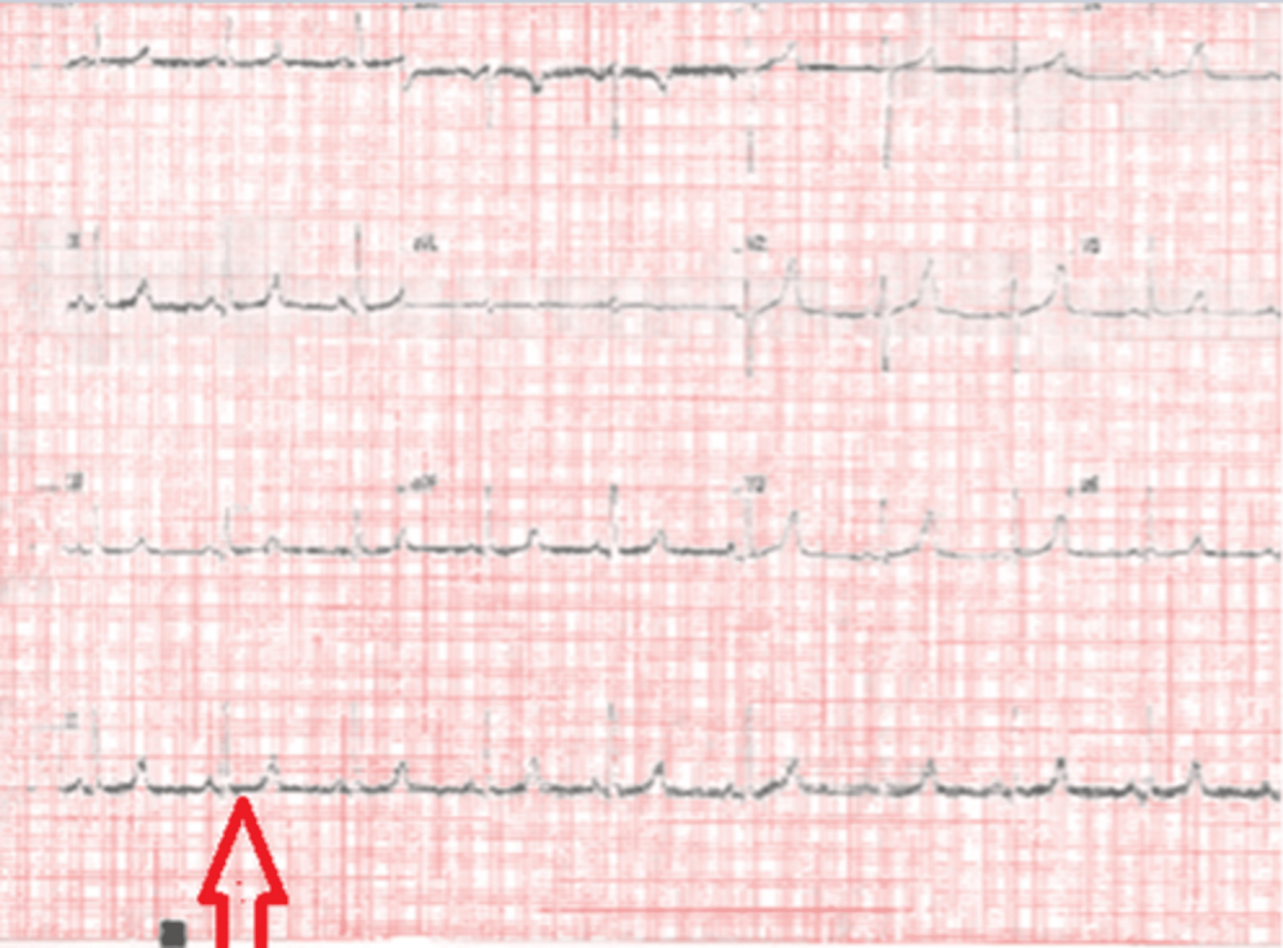
Prolonged QT interval (red arrow) on ECG prior to IV calcium therapy.

**Figure 3:**
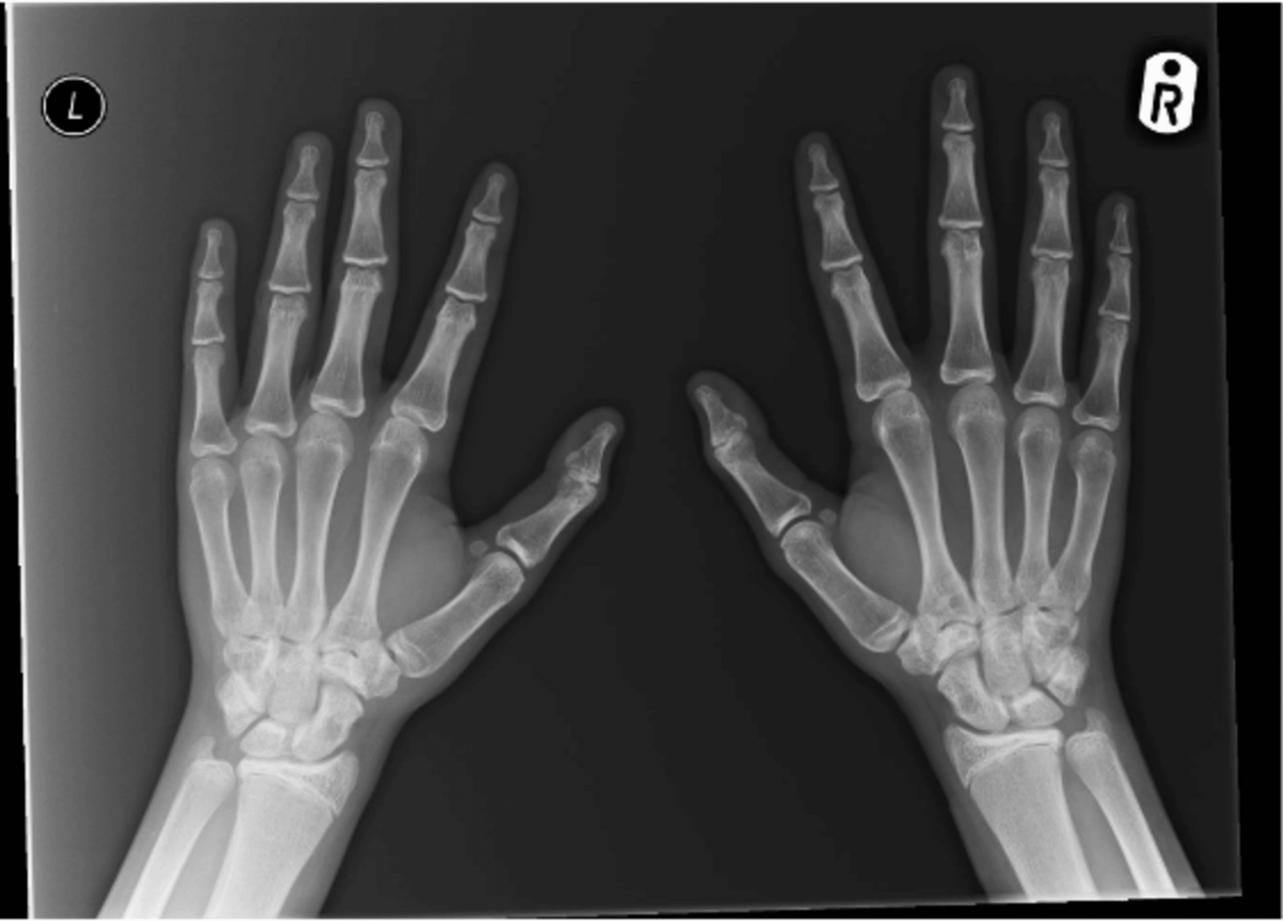
X-ray of the hands with no demonstrated shortening of the metacarpals, often seen in PHP. PHP: pseudohypoparathyroidism

During his admission, additional blood tests revealed severely low serum calcium (1.18 mmol/L), hypomagnesemia (0.57 mmol/L), elevated PTH (92.7 ng/L), and prolonged QT interval on electrocardiogram (ECG) (Figure 2). An X-ray of his hands did not show any shortening of the fourth or fifth metacarpal - a finding seen in PHP (Figure 3).

With these findings, the initial suspicion of a thiamine transporter or mitochondrial disorder was later morphed into a diagnosis of PHP. With treatment, his blood levels slowly improved (calcium 1.52 mmol/L and magnesium 0.70 mmol/L), and his prolonged QT on ECG had resolved. Symptomatically, he felt much better.

The genetic testing showed no mutation in the SLC19A3 gene, thereby ruling out thiamine transporter or mitochondrial disorder. The anterior pituitary hormones and nerve conduction studies were also unremarkable. These results, along with improvements in biochemistry, further supported the diagnosis of PHP. With this new diagnosis, he was referred to the adult endocrinology team and commenced 1-alfacalcidol 5 μg daily in divided doses. He was reviewed fortnightly, with serum calcium, PTH, and phosphate levels and renal function monitored. Good adherence to medication and stable blood levels led to annual reviews. He was eventually symptom-free, with stable blood levels at his most recent appointment in 2023. Relevant blood tests during his diagnostic journey are outlined in Table 1.

**Table 1:** Relevant blood results during the diagnostic process from initial presentation to the most recent endocrinology outpatient appointment.

Tests	Initial values	On admission (day 3)	Post admission (day 5)	Day 9	Five years later	Reference range
Serum corrected calcium	1.28 mmol/L	1.18 mmol/L	1.52 mmol/L	1.72 mmol/L	2.43 mmol/L	2.15-2.55 mmol/L
Serum magnesium	0.64 mmol/L	0.57 mmol/L	0.70 mmol/L	N/A	N/A	0.7-1.0 mmol/L
Serum PTH	N/A	92.7 ng/L	N/A	N/A	17.3 ng/L	15-65 ng/L

Interestingly, his younger brother, with a background of cerebral palsy and learning difficulties, was also diagnosed with PHP, presenting late at 17 years of age. He has had hemiplegia since birth and was also being managed by the orthopedic team for lower limb length discrepancies and fixed flexion deformities. He had a height of 1.91 m and utilized orthotic aids but no walking aids to mobilize.

He was not identified via genetic screening as he did not attend initially, but presented later on with symptomatic hypocalcemia. He similarly had a reduced corrected serum calcium of 1.7 mmol/L, an elevated PTH of 150 ng/L, and a normal vitamin D level. He was commenced on calcitriol and oral calcium and is under endocrinology follow-up. The younger sibling underwent genetic testing specifically to look for common mutations (GNAS, GNAS-AS1) found in PHP, which yielded results in keeping with PHP type 1b (heterozygous deletion of exons 5-7 of the STX16 gene and complete loss of the maternal methylation pattern at the GNAS A/B: TSS-differentially-methylated region (DMR), likely inherited from his maternal allele).

The older sibling is still due to undergo genetic testing, but a similar genetic defect is expected with loss of methylation in keeping with type 1b. Interestingly, there is no family history in the preceding generations of similar calcium metabolism disorders. Currently, both siblings are under regular follow-up with stable corrected calcium levels and no significant evidence of end-organ damage. Neither sibling has had any recent bone mineral density scan.

## Discussion

This case highlights the diagnostic challenges of pseudohypoparathyroidism (PHP), especially in patients with non-specific neurological symptoms such as fatigue, cramps, and gait disturbances. The initial suspicion of a metabolic or mitochondrial disorder was reasonable given the patient's symptoms, though it ultimately led to a delay in diagnosis. For these brothers, there were no recorded previous bone profile blood investigations prior to the elder brother’s admission with severe symptomatic hypocalcemia. PHP is a rare condition, often presenting with severe hypocalcemia symptoms [6]. In the literature, there are case reports of childhood seizures emerging due to PHP, secondary to cerebral calcifications [7].

Genetic testing is important, but a clear family history may not always be present. With more than 400 mutations in the GNAS region, patient categorization can be complex [1,6]. However, a consistent management goal is to maintain serum corrected calcium levels in the lower-normal range and to keep parathyroid hormone levels close to normal, through oral activated vitamin D, as effectively demonstrated in the present case [1,6].

A review of the literature found no universal guideline in the United Kingdom or the United States of America that recommends routinely assessing a child's bone profile solely on the basis of learning difficulties or congenital cerebral palsy. Due to the presence of brachydactyly, short stature, and other phenotypic features of AHO, earlier testing and recognition are usually the case in PHP type 1a and 1c, in which these features are present. As highlighted in Mantovani's study on the consensus diagnostic approach to PHP, the presence of AHO should prompt investigation with a full bone profile blood test, a vitamin D blood assay, parathyroid hormone levels, and skeletal imaging [1].

Due to the absence of the features of AHO in type 1b, the diagnosis proves more challenging, as highlighted in other case reports where it has been identified in late childhood, adolescence, or even adulthood [6,7]. The early acknowledgment of hypocalcemia as a potential cause for chronic muscle cramps, falls, and fatigue could lead to earlier diagnosis. Awareness of PHP can improve diagnosis and address complications, underscoring the need for interdisciplinary collaboration and thorough metabolic investigations in similar cases.

## Conclusions

In the present case, hypocalcemia was only identified as the cause of symptoms after nine months of symptomatic severe hypocalcemia. As outlined above, the absence of the typical features of AHO and the non-specific symptoms may have been responsible for this delay. Furthermore, although both siblings did have some learning difficulties, this was not necessarily due to the condition, and there is no consensus for routinely checking serum calcium levels in learning difficulties and cerebral palsy. We suggest that, based on our case and similar cases, the bone profile should be tested in younger patients presenting with complex neurological presentations and learning difficulties.

We also conclude that pseudohypoparathyroidism ought to be considered as one of the main differentials in the assessment of young patients presenting with unexplained neurological symptoms, learning difficulties, and hypocalcemia. An important learning point is the need to consider pseudohypoparathyroidism when intracranial calcifications are identified on brain imaging. This case highlights how seemingly unrelated symptoms involving multiple organ systems can converge on a single endocrine diagnosis. Early recognition and appropriate management with regular monitoring can lead to excellent long-term outcomes, as seen in the present report.
